# Spatio-Temporal Analysis of Forest Fire Risk and Danger Using LANDSAT Imagery

**DOI:** 10.3390/s8063970

**Published:** 2008-06-20

**Authors:** Bülent Sağlam, Ertuğrul Bilgili, Bahar Dinç Durmaz, Ali İhsan Kadıoğulları, Ömer Küçük

**Affiliations:** 1 Artvin Çoruh University, Faculty of Forestry, 08000 Artvin- Turkey; 2 Karadeniz Technical University, Faculty of Forestry 61080, Trabzon-Turkey; E-mails: bilgili@ktu.edu.tr (E.B.); b.dinc@ktu.edu.tr (B.D.D.); alikadi@ktu.edu.tr (A.I.K.); 3 Kastamonu University, Faculty of Forestry 37100, Kastamonu-Turkey; E-mail: omerkucuk@kastamonu.edu.tr (Ö.K.)

**Keywords:** forest fire management, fire risk, fire danger, GIS, landsat, spatial analysis

## Abstract

Computing fire danger and fire risk on a spatio-temporal scale is of crucial importance in fire management planning, and in the simulation of fire growth and development across a landscape. However, due to the complex nature of forests, fire risk and danger potential maps are considered one of the most difficult thematic layers to build up. Remote sensing and digital terrain data have been introduced for efficient discrete classification of fire risk and fire danger potential. In this study, two time-series data of Landsat imagery were used for determining spatio-temporal change of fire risk and danger potential in Korudag forest planning unit in northwestern Turkey. The method comprised the following two steps: (1) creation of indices of the factors influencing fire risk and danger; (2) evaluation of spatio-temporal changes in fire risk and danger of given areas using remote sensing as a quick and inexpensive means and determining the pace of forest cover change. Fire risk and danger potential indices were based on species composition, stand crown closure, stand development stage, insolation, slope and, proximity of agricultural lands to forest and distance from settlement areas. Using the indices generated, fire risk and danger maps were produced for the years 1987 and 2000. Spatio-temporal analyses were then realized based on the maps produced. Results obtained from the study showed that the use of Landsat imagery provided a valuable characterization and mapping of vegetation structure and type with overall classification accuracy higher than 83%.

## 1. Introduction

In the Mediterranean Basin fire plays a major role in many ecosystem processes. Recent statistics indicate that over 2,000 forest fires occur in Turkey every year, with an annual area burned ranging from 10 000 to 14 000 hectares [1]. To mitigate fire problem and minimize the threat of loss from wildfires, it is of crucial importance that forest managers conduct spatio-temporal analyses of forest fire danger and risk [2, 3]. Meanwhile, decision makers must also take into account the fire risk and danger potential that can lead to large scale severe forest fires as a result of forest growth [4, 5, 6], climatic change, land-cover (use) change [7, 8] and long-term fire suppression [9].

Fire risk and danger potential have generally been associated with stand fuel characteristics, topographical features and land use. These include fuel types, canopy closure, fuel characteristics over the stages of stand development, horizontal and vertical fuel (biomass) continuity, terrain structure and underlying landform, and the distribution of settlement and agricultural areas across the forest [10, 11, 12]. The spatio-temporal patterns of these characteristics are fundamental to fire risk and danger potential assessment [12-14]. Thus, it is extremely important to develop methods that can help managers accurately and timely assess fire danger potential [15] and predict the probability of fire risk on a spatio-temporal scale [16]. Conventional field measurements can be useful in this regard, and is still necessary for ground validation and local-scale applications, but these are extremely labour intensive, costly and difficult to extrapolate accurately over large areas. Satellite imagery and airborne sensors, on the other hand, have been successfully used for estimating, surveying and mapping forest fuels [2, 16-19] and assessing fire risk on large temporal and spatial scales [20] in a timely, easy and cost effective fashion.

At the landscape-level, remote sensing can support many aspects of fire management. Fuel and fire studies utilizing remotely sensed data such as Landsat, MODIS, SPOT and AVHRR can provide valuable information on fuel moisture [21], fuel characteristics [22, 23, 24], fire risk and danger, and fire frequency [15, 25, 26]. On the local scale, the use of Lidar [27, 28] and airborne hyperspectral sensors, such as multispectral infrared and visible imaging spectrometer (MIVIS) [18] and airborne visible/infrared imaging spectrometer (AVIRIS) data [2] allow the measurement of the three-dimensional structure of the canopy. These have been widely used to analyze vertical forest structure and to estimate critical parameters for fire behavior such as crown bulk density, tree height and basal area, [28-32]. In addition, using such airborne hyperspectral sensors, remote sensing techniques can provide new information concerning the canopy layer and vegetation parameters such as height, crown dimensions, volume and biomass [32-36] and detailed spatial information on forest attributes that may be relevant to spatial fire behavior models [32, 37].

The objective of this study is to determine forest stand parameters using remotely sensed data in a case study area in northwestern Turkey. The research focuses on classifying and mapping the stand parameters such as stages of stand development, crown closure, stand types, and land cover using the spatial analysis functions of GIS.

## 2. Study Area

**Figure 1.**
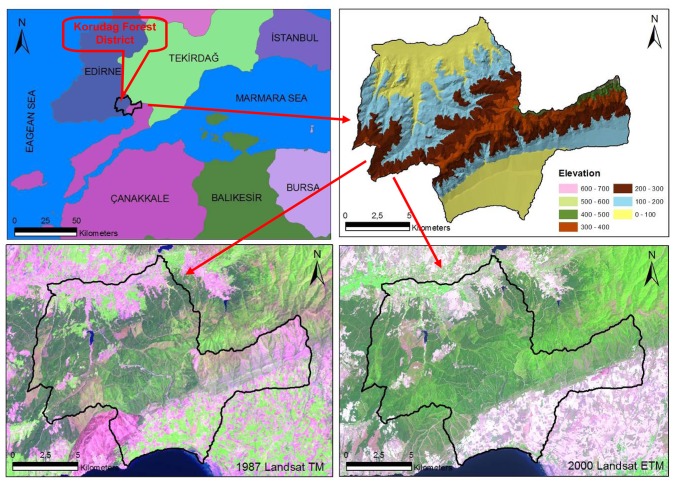
The geographic location of the study area.

Korudag Forest District, an area particularly vulnerable to forest fires due to its ecological characteristics and prevailing wind patterns, is a sub-temperate forest zone covering an area of 18,506 ha along the coast of the Saros Gulf in the north of Eagan Sea, northwestern Turkey (470600–492500 E, 4499750-4515600 N, UTM ED 50 datum Zone 35N) (Figure 1). The altitude varies between 0 and 700 m above sea level with an average slope of 12%. The vegetation is composed of calabrian pine (*Pinus brutia* Ten.), Anatolian black pine (*P. nigra* J.F. Arnold subsp.), *Quercus* spp. and shrubs.

The data used in this research are forest cover type maps of 1/25 000 scale for the years 1980, 1995 and 2004, a Landsat TM satellite image of 11.05.1987 and a Landsat ETM image of 25.07.2000. The forest cover types, used as ground truthing, were originally generated from both the stereo interpretation of black and white aerial photos with an average 1/25 000 scale and ground measurements with 300×300 sampling points. The Landsat images were interpreted with ERDAS image analysis program.

## 3. Materials and Methods

### 3.1. Geometric Correction of Landsat Images and Digitizing Stand Type Maps

Subsets of satellite images were rectified using 1/25,000 scale Topographical Maps with UTM projection (ED 50 datum) using first order nearest neighbour rules. A total of 20 ground points were used to register the ETM image with the rectification error of less than 1 pixel. The TM images, however, were registered to the already registered ETM images through image-to-image registration technique with rectification error of less than 0.5 pixels.

The forest stand type maps used in this research were first scanned, saved in tiff format and then registered to the digital topographic maps in the same manner as with the Landsat ETM image. Rectified forest stand type maps were digitized with a 1/3,000 to 1/5,000 screen view scale with Arc/Info 9.2™ GIS by a number of qualified foresters. This allowed the direct comparison of the features between the images and aerial photographs during the selection of sample plots to be used in image classification and accuracy assessment of classified images.

### 3.2. Image Classification of the 1987 Landsat TM and 2001 Landsat ETM Images

Ground reference data was obtained from more than 110 ground data points as signatures for each satellite image. The training points were equally distributed to each cover type with at least 10 points per cover type. For the supervised classification of the 1987 image, the stand type maps of 1980 and 1995 were combined to create ground signatures. Likewise, the stand type maps of 1995 and 2005 were combined to create ground signatures for the supervised classification of the 2000 image. These ground reference points were sampled on the cover type (stand) maps derived from the 2002 Ariel Photography and verified through ground measurements undertaken by the State Forest Management Teams in 2005. In order to classify cover types from the images, signatures were taken from the ground corrected stand type maps and adjusted based on the Transformed Vegetation Index, Principle Components Analysis-PCA and unsupervised classification image. Supervised maximum likelihood classification methods were employed in the analyses. Then the 1987 and 2000 images were checked for accuracy using ground data points that were not used in the original classification process together with other points of known condition, such as forest areas visually surveyed with binoculars, stand maps, urban areas and rock outcrops identified in the image. Equal Control Point methods were used in Erdas Imagine 9.0™ program with at least 30 points for each class [38]. The accuracy assessment of image was checked for each image and accepted if the accuracy was higher than %80. After the accuracy assessment, all images were clumped, eliminated 3×1 pixels and vectorized in Erdas Imagine 9.0™ program. These coverages were pre-processed to eliminate areas less than 0.3 ha for spatial landscape analysis with Fragstats™.

The Landsat TM image (1987) was successfully classified for nine fuel types, but regeneration areas and open areas were classified with a lower accuracy than other classes (69%, Table 1). However, this is generally acceptable as the overall classification accuracy is much higher (83%) with the Kappa statistics (Conditional Kappa for each Class) value of 0.812.

The Landsat ETM image (2000) was classified into 11 fuel type classes, which differed from Landsat TM because of the changing land cover classes over time, and better distinguished the mixed land cover class (ÇzMab3) with the kappa statistics value of 0.888. However, regeneration and open area classes were not distinguished successfully from each other (69%, Table 2). Notwithstanding this, Landsat ETM classification is generally acceptable due to a higher overall classification accuracy of 86% and Kappa statistics (Conditional Kappa for each Class) value of 0.853.

### 3.3. Determination of fire risk and danger potential indices

Fire risk refers to the probability of ignition, as determined by the presence and activity of causative agents (i.e., man, lightning, etc) [39-44]. The term “danger” refers to sum of constant and variable factors affecting the ignition, spread, and resistance to control, and subsequent fire damage [45]. The risk and danger potential of wildland fires must be determined and mapped both spatially and temporally [39]. Fire risk and danger potential (FRDP) maps are digital cartography of fire ignition and severity and are based on stand characteristics, topographic features and land use practices in a specific region. These maps are developed through incorporating satellite and surface observations in an index that correlates well with fire ignition and danger.

**Figure 2.**
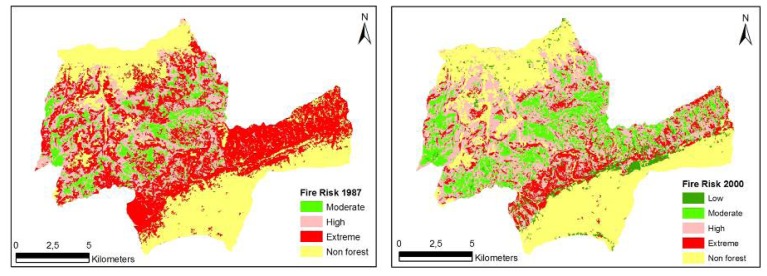
Spatial fire risk maps for Korudag Forest District derived from the 1987 - 2000 Landsat images.

Fire risk potential index was determined based on land use attributes such as settlement areas and agricultural lands together with species composition, slope and insolation (Table 3). Following steps were taken in the process. First, each variable class was assigned a fire risk rating (extreme, high, moderate or low) according to the risk potential of each class. Second, each fire risk class was rated on a scale from 1 to 5 (Table 3). Third, all variables (layers) were then integrated through GIS using the equation generated. The equation used was of the form [46]:

*FRI* = 10*SC_i_* + 2*AL_j_* + 2*SA_k_* + 3*S_l_* + 2*IS_m_*

where FRI is the relative numerical rating of fire risk; SC, species composition (5 classes); AL, proximity of agricultural lands to forest (4 classes); SA, proximity to settlement areas (4 classes); S, slope factor (4 classes); and IS, insolation factor (9 classes). The subscripts *i*, *j*, *k*, *l*, *m* indicate subclasses determined by the fire risk potential. Finally, criterion-based analysis (Table 4) [46] was carried out to create fire risk maps showing different categories for 1987 and 2000 (Figure 2).

**Figure 3.**
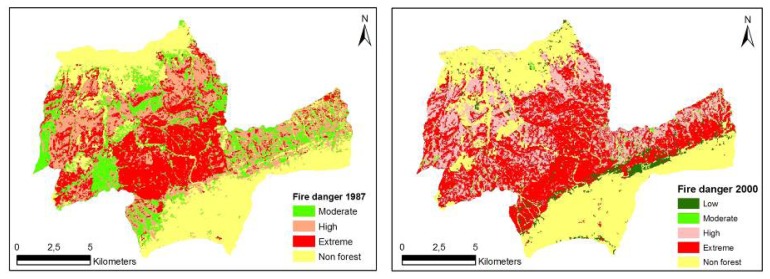
Spatial fire danger maps for Korudag Forest District derived from the 1987-2000 Landsat images.

Fire danger potential index was determined based on species composition, stages of stand development, stand crown closure and topographic features such as insolation and slope (Table 5). Following steps were taken in the process. First, each variable class was assigned a fire danger rating (extreme, high, moderate or low) according to the danger potential of each class. Second, each fire danger class was rated on a scale from 1 to 5 (Table 5). Third, all variables (layers) were then integrated through GIS using the equation generated. The equation used was of the form:

$$\text{FDI}=SC_{i}^{2}(CC_{n}+SD_{p}+S_{l}+IS_{m})$$

where FDI is the relative numerical rating of fire danger; SC, species composition (5 classes); CC, stand crown closure (5 classes); SD, stages of stand development (6 classes); S, slope factor (4 classes); and IS, insolation factor (9 classes). The subscripts *i*, *n*, *p*, *l*, *m* indicate subclasses determined by the fire danger potential.Unlike the FRI, a different approach was employed here in developing the relationship used. Species composition was weighted (squared) and incorporated into the relationship as a multiplier so as to eliminate incorrect classification and obtain a wider range of values. This approach avoids the limitation of a simple additive model in which incorrect values could be obtained irrespective of species composition. Species composition is one of the most important factors affecting fire danger potential. Finally, criterion-based analysis (Table 6) was carried out to create fire danger maps showing different categories for 1987 and 2000 (Figure 3).

As a result, FRDP maps were obtained using seven factors, namely slope, insolation, stages of stand development, species composition, crown closure, proximity of agricultural lands to forest and distance from settlement areas (Tables 3 and 5). The factors used in the calculation of the FRI and FDI were selected based on experience and relevant literature.

Slope does not necessarily have an effect on the probability of an ignition but has a strong effect on fire behavior [46]. Forest stands on steeper slopes have greater fire danger. Slope was taken as the mean percent slope for each polygon (Tables 3 and 5). Insolation was taken as the mean aspect for each polygon. Southern and southwestern exposures in the northern hemisphere have the greatest fire danger (Tables 3 and 5). Stages of stand development are a measure of forest structure. The accumulation of crown and surface fuels increases with stand age and development [47, 48]. Forest structure and stand fuel characteristics (fuel loading and continuity) can dramatically change fire danger. The highest fire danger usually occurs in the pole/very young and young stages of stands due to lower crown base height and increased vertical fuel continuity [48-50]. The stages of stand development considered in this study involved non-forested/newly planted, shrub/herb (1±15 years), pole/very young (15±30 years), young (31±60 years), mature (61±100 years), and old forest (101 years or greater) (Table 5).

Relative species composition is an indicator of site conditions and directly affects flammability of the fuel complexes. Deciduous forest stands are considered to represent low fire danger areas, whereas coniferous (usually pine) stands are generally associated with high fire danger. Mediterranean shrubs are also known to have high fire danger potential due to the high flammability of the fuels and fast spread of fires in these vegetation types (Tables 3 and 5). Crown closure provides an indicator of the ease with which fire can spread. The higher the crown closure, the more intense the fires burn [48]. Crown closure was taken as the mean percent cover for each polygon (Table 5). In addition, distances from settlement areas and proximity of agricultural lands to forest were used to provide an assessment of fire risk together with other factors. Both factors are a measure of the extent to which human activities contribute to fire risk (Table 3). All variables included in the analyses were rated on a scale from 1 to 5.

As a last step, Fragstats™ [51] was used to quantify landscape structure, fire risk and danger potential of Korudag Forest District for each of the land use classes. Forest stand attributes were classified to easily compare the accuracy of the satellite images and determine the changes in landscape structure at spatio-temporal scale (Table 7).

Fragstats calculates a number of spatial metrics for each patch and cover class as well as for the entire landscape. Selected metrics were analyzed for the land use class for the study area in 1987 and 2000. The metrics were: class Percent of Landscape (PL), Number of patches (NP), Largest Patch Index (LPI), Mean Patch Size (MPS) and Area Weighted Mean Shape Index (AWMSI).

## 4. Results

Changes in landscape structure and associated fire risk and danger potential at spatio-temporal scale are presented for the Korudag Forest District. According to the stand type maps developed for the Korudag Forest District, the district has a total of 18.506 ha land area. The analyses of the 1987 and 2000 Landsat images showed there was a slight decrease in the forested areas. The percentage of the forest cover in the study area was 67.7% in 1987 and 63.9% in 2000 (Table 8), indicating a cumulative forest disturbance of 3.8% (703 ha) in the district as a whole and 5.6 % in the forested areas of the district, representing an annual rate of forest disturbance of 0.44%. The decrease in forest cover was a result of several factors including degraded regeneration and plantation areas, mature stands harvested and areas burned immediately prior to 2000, and a slight increase in the settlement areas. Evaluations concerning the crown closure in the area showed that medium to fully covered areas increased by 1.371 ha and sparsely covered regeneration areas decreased by 2.672 ha. This could mainly be attributed to the growth and development of forest stands.

The spatial structure of fire risk maps developed for the study area for the years 1987 and 2000 was evaluated based on forest characteristics and indices generated by Fragstats. The evaluations were based on the Number of Patches (NP), Area-weighted Mean Shape Index (AWMSI), Mean Patch Size (MPS) and Largest Patch Index (LPI). The structure of fire risk maps developed for the study area for the years 1987 and 2000 were quantified respectively as NP (8.902, 12.984), AWMSI (9.70, 7.72), MPS (2.08 ha, 1.43 ha) and LPI (20.27, 18.96) (Table 10). The results indicated that there was a substantial decrease in the extreme fire risk class, but an increase in the high, moderate and low fire risk classes over the 13 years. The decrease in the extreme fire risk class can be explained by the causative agents that relate to fire risk. Fire risk is associated with ignition, and ignition in the study area is mostly triggered by human activities. Therefore, the forest openings present in the area and relatively free access to these areas by the local people in 1987 would increase the probability of ignition. However, through the natural development and growth, and expansion of stands through new plantations, landscape fragmentation decreased and the activities of local people were limited to mostly the unforested areas, which, in turn, decreased the extreme fire risk potential (Table 10, Figure 2).

The spatial structure of fire danger maps developed for the study area for the years 1987 and 2000 was evaluated based on forest characteristics and indices generated by Fragstats. The evaluations were based on NP, AWMSI, MPS and LPI. The structure of fire danger maps were quantified respectively as NP (11.651, 9.753), AWMSI (6.48, 11.13), MPS (1.59 ha, 1.90 ha) and LPI (16.95, 18.96) (Table 9). Over the 13 years, while the “extreme” fire danger class remained relatively the same (280 ha increase), “high” fire danger class increased noticeably by 1.840 ha. On the other hand, “moderate” and “low” fire danger classes decreased. Any change in fire danger classes can be explained by the factors that relate to fire danger. Fire danger is associated mostly with the conditions of the fuels together with the factors affecting the ignition, spread, resistance to control, and subsequent fire damage. In 1987, forest landscape in the study area was highly fragmented and forest stands were relatively young, causing fuel load and continuity to be low. As the stands grew older and forest gaps closed over the time, fuel continuity and availability increased thereby increasing the fire danger in general. The decrease in the “moderate” and “low” fire danger classes is simply the result of these classes moving up and becoming a part of “high” and “extreme” fire danger classes. (Table 8, Figure 3).

## 5. Discussion

Landsat images have been successfully used to estimate forest cover and vegetation types in a wide range of forest ecosystems [e.g, 7, 8, 15]. The national fire management and science communities require remote-sensing mapping and characterization of vegetation structure because remotely sensed data is relatively reliable, timely and cost effective. In this research, Landsat data were analysed for a test area in northwestern Turkey to ascertain how well remote sensing data could be used to characterize and evaluate the spatio-temporal change in fire risk and danger potential.

Results from the analyses showed that the spatial and spectral resolution of Landsat imageries could provide a valuable characterization of fuel types with the classification accuracy being higher than 83% (Tables 1 and 2). The structure and composition of forested areas in the study area changed considerably from 1987 to 2000 (Figure 2), resulting in the increasingly dense and connected fuels, continuous canopy fuel layer and low canopy base height [48-50]. In the study area, fire exclusion and planting activities coupled with forest growth and development had caused the accumulation of vegetation and fuel, greater continuity in vertical and horizontal stand structure and, thus, contributed to the increase in fire danger potential between 1987 and 2000. However, there was a relative decrease in fire risk in 2000 compared with that in 1987. This can be attributed to the fact that forest gaps open to free access by people closed up as a result of the forest encroachment into open fields, resulting in a relative decrease in fire risk, but an increase in fire danger.

Increased fire danger potential is clear evidence that high intensity and high severity wildland fires can readily occur and affect larger areas. To reduce these disturbances, forest managers must prioritize areas for fire mitigation and hazard (fuel) reduction. The results indicate that if the alterations that take place at the stand level, due to stand development and silvicultural activities, are intensive; spatial variability is also high. Therefore, forest managers should consider prescribed burning, silvicultural treatments (e.g. thinning and pruning) and construction of fire and fuel breaks to reduce landscape fuels and interrupt fuel continuity.

Fuel management activities, thinning and prescribed burning, have been repeatedly shown to reduce fire intensities and increase survival of some forest types [52-54], thereby reducing the negative impacts of fires and providing benefits in the form of additional fuel management and ecological process in forest ecosystems [55]. Evidence exists that fire occurrence too may be reduced by prescribed burning [52, 56, 57] and that the spatial patterns of fuel treatments can theoretically alter the growth and development rate of large fires [58, 59]. The partitioning of the blocks of adjacent and neighbour large areas which have the high fire risk and danger potential is crucial for fire prevention and suppression activities. Thus, these applications should be integrated into regular fire and forest management plans.

Forest management has evolved around a wide range of production objectives (e.g. timber), environmental concerns (e.g. biodiversity) and non-market functions (e.g. protection and recreation), but rarely have the wildfire concerns been incorporated into forest management planning. Fire is an integral part of many forest ecosystems, and the role that fires play in ecosystem dynamics and land uses is of crucial importance in setting up management objectives. However, difficulties in measurement and quantification of these criteria sometimes lead to either descriptive approaches to the problem or total exclusion from forest management planning [60]. The results from this study may have important implications for fire and forest management planning in areas where fire is both a threat to and an integral part of many forest ecosystems.

## Figures and Tables

**Table 1.** Confusion matrix for the 1987 Landsat TM image supervised classification.

Classes*	**Fuel Type (FT)**	**Reference Totals**	**Classified Totals**	**Number Correct**	**Accuracy (%)**		**Kappa**

					**Producers**	**Users**	
Open Areas	FT8	39	30	27	69.23	90.00	0.883
Water/lake	FT10	30	30	30	100.00	100.00	1.000
Çz*a*0-*a*1	FT1-_1_	27	30	25	92.59	83.33	0.815
Çz*ab*3	FT2	30	30	28	93.33	93.33	0.925
Çz*b*3-*bc*3	FT4-_1_	37	30	26	70.27	86.67	0.846
Çz*c*3-*cd*3-*d*3	FT5	27	30	25	92.59	83.33	0.815
Çk*bc*3	FT4-_2_	26	30	19	73.08	63.33	0.594
Çk*c*2-*c*3	FT6	29	30	22	75.86	73.33	0.701
Settlement	FT9	24	30	23	95.83 assification Acc	76.67	0.744

Overall Kappa Statistics = 0.813				Overall Classification Accuracy = 83.33%			

* Çz, Calabrian pine; Çk, Anatolian black pine. *a*, *b*, *c* and *d* are indicators for the stages of stand development (*a*, regeneration; *b*, regeneration-young, *c*, mature; *d*, old-mature). 0,1,2,3 are indicators of crown closure (0, no crown closure; 1, low crown closure (1-11%); 2, moderate crown closure (12-40%); 3, high crown closure (>70%)).

**Table 2.** Confusion matrix for the 2000 Landsat ETM image supervised classification.

Classes*	**Fuel Type (FT)**	**Reference Totals**	**Classified Totals**	**Number Correct**	**Accuracy (%)**		**Kappa**

					**Producers**	**Users**	
Open Areas	FT8	42	30	29	69.05	96.67	0.962
Water/Lake	FT10	30	30	30	100.00	100.00	1.000
Çzcd3, c3	FT5	36	30	27	75.00	90.00	0.888
Çzab3	FT2	29	30	24	82.76	80.00	0.781
Çza1	FT1-_2_	25	30	24	96.00	80.00	0.784
Çkcd3, c3	FT6	28	30	26	92.86	86.67	0.854
Çzbc3	FT4-_1_	28	30	26	92.86	86.67	0.854
ÇzMab3, ab2	FT3	35	30	27	77.14	90.00	0.888
Çza0	FT1-_1_	27	30	24	88.89	80.00	0.782
BDy	FT7	28	30	27	96.43	90.00	0.891
Settlement	FT9	22	30	22	100.00	73.33	0.714

Overall Kappa Statistics = 0.853				Overall Classification Accuracy = 86.67%			

* Çz, Calabrian pine; Çk, Anatolian black pine; M, Mediterranean scrubs; BDy, Degraded deciduous species;. *a*, *b*, *c* and *d* are indicators for the stages of stand development (*a*, regeneration; *b*, regeneration-young, *c*, mature; *d*, old-mature). 0,1,2,3 are indicators of crown closure (0, no crown closure; 1, low crown closure (1-11%); 2, moderate crown closure (12-40%); 3, high crown closure (>70%)).

**Table 3.** Weights and ratings assigned to variables and classes for fire risk.

**Variables**	**Classes**		**Value Assigned**	**Fire Risk**
**Species composition (weight = 10)**	(1) Calabrian pine		5	Extreme
	(2) Calabrian pine + black pine		5	Extreme
	(3) Shrub		4	High
	(4) Degraded areas		2	Moderate
	(5) Oak + Coppice		1	Low

**Slope (weight = 3)**	(6) 0 – 5 %		1	Low
	(7) 5 – 15 %		2	Moderate
	(8) 15 – 35 %		3	High
	(9) > 35 %		5	Extreme

**Insolation (weight =2)** 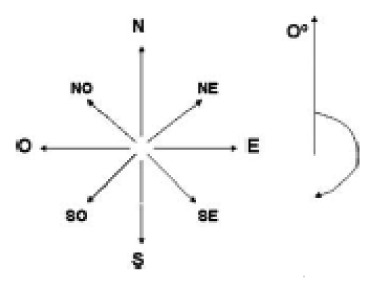	(10) 0- 23	N	1	Low
	(11) 23- 68	NE	2	Moderate
	(12) 68 -113	E	2	Moderate
	(13) 113 – 158	SE	3	High
	(14) 158 – 203	S	5	Extreme
	(15) 203 – 248	SW	5	Extreme
	(16) 248 – 293	W	2	Moderate
	(17) 293 – 338	NW	2	Moderate
	(18) 338 – 360	N	1	Low

**Proximity of Agricultural Lands to Forest (m) (weight = 2)**	(19) 0-100		5	Extreme
	(20) 100-200		3	High
	(21) 200-300		2	Moderate
	(22) 400 <		1	Low

**Proximity to Settlement Areas (m) (weight = 2)**	(23) 0-100		5	Extreme
	(24) 100-200		3	High
	(25) 200-300		2	Moderate
	(26)400 <		1	Low

* non-vegetated sites were assigned a “0” fire risk rating

**Table 4.** Criterion-based analysis for fire risk mapping.

Species Composition (SC)	Proximity of Agricultural Lands To Forest (AL)	Distance From Settlement Areas (SA)	Slope (%) (S)	Insolation (°) (IS)	Fire Risk Class	Fire Risk Index (FRI)
E_SC_	E_S_	E_IS_	E_AL_	E_SA_	Extreme	95
E_SC_	H_S_	H_IS_	H_AL_	H_SA_	Extreme	77
E_SC_	H_S_	H_IS_	M_AL_	M_SA_	Extreme	73
E_SC_	H_S_	H_IS_	M_AL_	L_SA_	High	71
H_SC_	H_S_	H_IS_	M_AL_	M_SA_	High	63
H_SC_	H_S_	H_IS_	M_AL_	L_SA_	Moderate	61
M_SC_	M_S_	M_IS_	M_AL_	M_SA_	Moderate	48
M_SC_	M_S_	M_IS_	M_AL_	L_SA_	Low	46
L_SC_	L_S_	L_IS_	L_AL_	L_SA_	Low	19

Subscript: E; extreme, H; high, M; moderate, L; low

**Table 5.** Ratings assigned to variables and classes for fire danger.

**Variables**	**Clases**		**Value Assigned**	**Fire Danger**
**Species composition**	(1) Calabrian pine		5	Extreme
	(2) Calabrian pine + black pine		5	Extreme
	(3) Shrub		4	High
	(4) Degraded areas		3	Moderate
	(5) Oak + Coppice		1	Low

**Stand crown closure (%)**	(6) Bare Land		1	Low
	(7) <11%		1	Low
	(8) 11-40%		2	Moderate
	(9) 41-70%		3	High
	(10) 71%>		5	Extreme

**The stage of stand development**	(11) (a) newly planted -average dbh: < 8 cm		2	Low
	(12) (a) regenerated and (b) young - average dbh: < 0 – 8 and 8 – 19.9 cm		5	Extreme
	(13) (b) young - average dbh: 8 – 19.9 cm		5	Extreme
	(14) (b) young and (c) mature – average dbh: 8 – 19.9 cm and 20 – 35.9 cm		4	Moderate
	(15) (c) mature - average dbh: 20 – 35.9 cm		3	Moderate
	(16) (c) mature and (d) overmature - 20 – 35.9 cm >36 cm		2	Low
	(17) (d) overmature - average dbh: >36 cm		1	Low

**Slope**	(18) 0 – 5 %		1	Low
	(19) 5 – 15 %		2	Moderate
	(20) 15 – 35 %		3	High
	(21) > 35 %		5	Extreme

**Insolation** 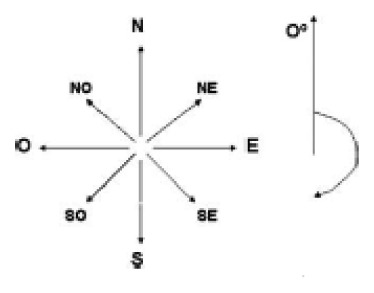	(22) 0- 23	N	1	Low
	(23) 23- 68	NE	2	Moderate
	(24) 68 -113	E	2	Moderate
	(25) 113 – 158	SE	3	High
	(26) 158 – 203	S	5	Extreme
	(27) 203 – 248	SW	5	Extreme
	(28) 248 – 293	W	2	Moderate
	(29) 293 – 338	NW	2	Moderate
	(30) 338 – 360	N	1	Low

* non-vegetated sites were assigned a “0” fire danger rating

**Table 6:** Criterion-based analysis for fire danger mapping.

Species Composition (SC)	Stand Crown Closure (%) (CC)	Stages of Stand Development (SD)	Slope (%) (S)	Insolation (°) (IS)	Fire Danger Class	Fire Danger Index (FDI)
E_SC_	E_CC_	E_SD_	E_S_	E_IS_	Extreme	500
E_SC_	H_CC_	H_SD_	E_S_	E_IS_	Extreme	425
H_SC_	E_CC_	E_SD_	E_S_	E_IS_	Extreme	320
E_SC_	H_CC_	H_SD_	H_S_	H_IS_	Extreme	300
E_SC_	H_CC_	E_SD_	L_S_	L_IS_	Extreme	275
H_SC_	H_CC_	H_SD_	E_S_	E_IS_	High	272
H_SC_	H_CC_	H_SD_	H_S_	H_IS_	High	208
H_SC_	H_CC_	M_SD_	H_S_	H_IS_	High	192
M_SC_	E_CC_	E_SD_	E_S_	E_IS_	Moderate	180
H_SC_	M_CC_	M_SD_	M_S_	M_IS_	Moderate	144
H_SC_	M_CC_	L_SD_	M_S_	M_IS_	Moderate	128
M_SC_	E_CC_	E_SD_	M_S_	M_IS_	Moderate	126
E_SC_	L_CC_	L_SD_	L_S_	L_IS_	Low	125
L_SC_	L_CC_	L_SD_	L_S_	L_IS_	Low	4

Subscript: E; extreme, H; high, M; moderate, L; low

**Table 7.** Fuel type and vegetation typologies.

Fuel types (FT)	Species composition	Structural state	Crown closure
Fuel type 1a	Calabrian pine	Newly planted	No crown closure
Fuel type 1b	Calabrian pine	Regeneration	New crown closure (1-11%)
Fuel type 2	Calabrian pine	Regeneration-young	High (>70%)
Fuel type 3	Calabrian pine-shrubs	Regeneration-young	High (>70%)
Fuel type 4a	Calabrian pine	Young	High (>70%)
Fuel type 4b	Anatolian black pine	Young- mature	High (>70%)
Fuel type 5	Calabrian pine	Old-mature	High (>70%)
Fuel type 6	Anatolian black pine	Mature state	Moderate and High (40-100%)
Fuel type 7	Degraded deciduous	All states	Low (0-10%)
Fuel type 8	Open area	Roads, bare soils	No vegetation cover
Fuel type 9	Settlement	Settlement areas	Houses, buildings, farmings
Fuel type 10	Water	Lakes, natural waters	

**Table 8.** The transition matrix of fuel type change in Korudag Forest District (using the 1987 and 2000 Landsat Images).

**1987-2000**	**FT1a**	**FT1b**	**FT2**	**FT3**	**FT4a**	**FT5**	**FT6**	**FT7**	**FT8**	**FT9**	**FT10**	**Totals (1987)**
**FT1a**	690.51	743.8	367.14	896.08	1059.6	140.19	168.73	368.31	1056.46	10.54	5.59	5506.95
**FT2**	5.5	35.76	191.55	32.26	117.62	23.27	78.53	2.13	4.94			491.56
**FT4a**	146.27	239.21	370.12	122.31	983.14	456.93	357.45	58.36	164.74	0.04	0.15	2898.72
**FT4b**	10.84	34.15	59.56	9.24	52.23	78.51	85.42	0.1	11.06			341.11
**FT5**	244.66	254.06	70.85	33.63	256.44	1134.17	241.44	13.22	158.05		0.62	2407.14
**FT6**	35.84	122	90.25	50.11	152.94	200.98	199.68	3.42	24.79			880.01

**FT8**	139.36	133.13	7.47	150.8	120.14	24.36	6.81	149.06	4993.85	96.67	5.64	5827.29
**FT9**	0.42	0.12						2.38	83.45	46.21	0.07	132.65
**FT10**	0.06				0.2				5.07		15.28	20.61

**Totals (2000)**	1273.46	1562.23	1156.94	1294.43	2742.31	2058.41	1138.06	596.98	6502.41	153.46	27.35	18506.04

**Table 9.** Change of Fire Danger Pattern in Korudag Forest District derived from the 1987-2000 Landsat Images.

	Fire Danger					

	Low	Moderate	High	Extreme	Non Forest	Landscape

Class Area (ha)	597.0	3068.7	4728.5	4728.2	5980.5	18506.0
		208.7	4448.3	6568.7	6683.3	18506.0

Number of Patches	838	3142	4599	2799	1111	11651
		1195	4367	2708	645	9753

Mean Patch Size (ha)	0.71	0.98	1.03	1.69	5.38	1.59
		0.17	1.02	2.43	10.36	1.90

Percent of Landscape (%)	3.23	16.58	25.55	25.55	32.32	100.00
		1.13	24.04	35.49	36.11	100.00

Largest Patch Index (%)	0.29	1.60	1.54	7.37	16.95	16.95
		0.02	2.22	18.62	18.96	18.96

Patch density (number of patches per 100 ha)	4.53	16.98	24.85	15.12	6.00	62.96
		6.46	23.60	14.63	3.49	52.70

Patch size coefficient of variation (%)	404.32	829.73	847.36	2088.91	2051.11	2446.33
		182.85	921.54	2788.64	1588.49	2936.67

Area-weighted Mean Shape Index	2.26	4.02	4.79	8.05	7.83	6.48
		1.47	5.71	19.81	7.30	11.13

**Table 10.** Change of Fire Risk Pattern in Korudag Forest District derived from the 1987-2000 Landsat Images.

	Fire Danger					

	Low	Moderate	High	Extreme	Non Forest	Landscape

Class Area (ha)	709.6	1604.7	4064.8	6855.9	5980.5	18506.0
		2960.0	5844.2	2309.0	6683.2	18506.0

Number of Patches	1526	1755	3980	2055	1112	8902
		3218	4579	3017	644	12984

Mean Patch Size (ha)	0.46	0.91	1.02	3.34	5.38	2.08
		0.92	1.28	0.77	10.38	1.43

Percent of Landscape (%)		8.67	21.96	37.05	32.32	100.00

	3.83	16.00	31.58	12.48	36.11	100.00

Largest Patch Index (%)	0.29	0.51	1.86	20.27	16.95	20.27
		1.06	7.94	1.85	18.96	18.96

Patch density (number of patches per 100 ha)	8.25	9.48	21.51	11.10	6.01	48.10
		17.39	24.74	16.30	3.48	70.16

Patch size coefficient of variation (%)	465.21	566.41	831.70	2496.15	2052.03	2704.35
		815.00	1867.97	928.94	1587.27	2784.17

Area-weighted Mean Shape Index	2.16	3.35	5.20	15.49	7.83	9.70
		4.64	11.50	5.01	7.30	7.72
